# Transferring Surgical Expertise: Analyzing the Learning Curve of Robotic Cardiac Surgery Operative Time Reduction When Surgeon Moves from One Experienced Center to Another

**DOI:** 10.3390/jcdd11030081

**Published:** 2024-02-29

**Authors:** Sherif M. Khairallah, Mohamed Rahouma, Stephanie L. Mick

**Affiliations:** 1 Cardiothoracic Surgery Department, Weill Cornell Medicine New York-Presbyterian Hospital (WCM), 525 East 68th Street, Suite M404, New York, NY 10065, USA; smk4005@med.cornell.edu; 2 National Cancer Institute, Cairo University, Cairo 11562, Egypt

**Keywords:** learning curve, robotic cardiac surgery, surgeons transfer

## Abstract

Background: Robotically assisted cardiac surgery is performed in a team setting and is well known to be associated with learning curves. Surgeon and operative team learning curves are distinct entities, with total operative time representing the entire operative team (surgery, anesthesia, nursing, and perfusion) and cross-clamp time representing mainly the surgical team. Little is known about how a team learning curve evolves when an experienced surgeon transitions from one surgical center to another. This study investigates the dynamics of the team learning curve expressed as total operative time in the case of a surgeon with previous experience transitioning to a new team. Methods: A retrospective analysis was conducted on robotic cardiac surgeries performed by a surgeon who transitioned from one experienced surgical center to another. Operative time data were collected and categorized to assess the evolution of the learning curve. Statistical analysis, including learning curve modeling and linear regression analysis, was used to evaluate changes in total time in the operating room per case. Results: 103 cases were included in Weill Cornell Medicine (2019–2023). The median patient age was 63 years, 68% were males, 90.3% of cases were repaired for degenerative mitral valve disease, and the median body mass index was 23.87. Operative time (ORT) decreased from a median of 5.00 h [95%CI: 4.76, 6.00] in the first 30 cases to 4.83 [95%CI: 4.10, 5.27] thereafter, with the apparent curve plateauing indicative of the adaptation period to the new surgical environment (*p* = 0.01). Subgroup analysis among mitral cases (*n* = 93) showed a decrease in ORT from 5.00 [95%CI: 4.71, 5.98] in the first 26 cases to 4.83 [95%CI: 4.14, 5.30] (*p* = 0.045). There was no difference between the initial 30 cases and subsequent cases regarding cardiopulmonary bypass time, myocardial ischemia time, reoperation for bleeding, prolonged ventilation, reintubation, renal failure, need for an intra-aortic balloon pump, readmission to the ICU, reoperation for valvular dysfunction within 30 days, pneumonia, and deep venous thrombosis. Multivariate significant predictors of longer operative time were the first 30 cases, resection-based repairs, and MAZE as a concomitant procedure. Conclusions: Total operative time can be expected to decrease after about 30 cases when an experienced robotic surgeon moves between centers. Complications and cross-clamp times are less susceptible to a learning curve phenomenon in such a circumstance, as these depend primarily on the operating surgeon’s level of experience. Understanding these dynamics can inform the planning and management of surgical transitions, ensuring optimal patient care and continued improvement in surgical outcomes.

## 1. Introduction

Mitral valve repair is widely recognized as being superior to replacement in terms of long-term durability and reduced complications for treating degenerative mitral regurgitation [1,2]. The choice between replacement and repair is predominantly dependent on the surgeon’s experience and volume [3,4,5,6]. For instance, New York State data have demonstrated that surgeons with lower annual volumes (performing fewer than 25 operations per year) were more likely to opt for mitral replacement rather than repair in patients with degenerative disease, and patients undergoing surgery by lower-volume surgeons demonstrated worse survival rates and higher rates of reoperation in the long term compared to those undergoing surgery by higher-volume practitioners. Interestingly, this study also found that the presence of a high-volume surgeon at the same institution was linked to improved repair rates for low-volume surgeons at the same centers [7].

The use of robotics in mitral valve surgery started in 1998 [8], with the first complete repair performed by the East Carolina University group in 2000 [9]. Adoption of this new, expensive, and technically demanding procedure has been slow due to concerns about the time and effort required for efficient, safe, and effective use by both surgeons and health systems. Over the years, numerous studies have shown that robotic mitral valve surgery is equivalent in safety, outcomes, and repair rates compared to conventional sternotomy surgery. The benefits of robotic surgery included improved cosmetic results, a shorter hospital length of stay, a faster return to normal activities, reduced transfusion rates, and the ability to afford the surgeon an excellent 3D view of the mitral valve with a 10× magnification.

As with any technique, the use of robotics in mitral valve surgery is subject to a learning curve. The primary concern during this process is ensuring patient safety and maintaining clinical effectiveness during the early stages of the learning curve. Several studies have investigated the learning curve in robotic mitral surgery, but the data are limited. Some studies have reported a rapid decrease in operative time, composite complication rates, and increased efficiency as surgeons and surgical teams become more experienced [10,11,12].

Overall, the available evidence suggests that the learning curve in robotic mitral surgery is complex and dependent on various factors, including the surgeon’s prior experience, the surgical team’s experience (consisting of cardiac anesthesia, surgical assistants, surgical nurses, and technicians), the patient population, and the specific surgical techniques used. Further research is needed to fully understand the impact of experience on outcomes in robotic mitral surgery. Herein, we carried out this investigation to assess the learning curve of robotic mitral surgery at the Weill Cornell Medical Center—New York Presbyterian Hospital, a center of excellence and a leading tertiary referral center.

## 2. Patients and Methods

### 2.1. Inclusion, and Exclusion Criteria

The study included all patients between June 2019 and December 2022 who had isolated moderately severe or severe primary degenerative mitral regurgitation affecting either the anterior and posterior leaflets or bileaflet and required repair according to the patient selection protocol of Weill Cornell Medicine and underwent robotic repair. Supplementary Figure S1 showed the robotic team ergonomics at Weill Cornell Medicine. Patients with cardiac tumors who underwent robotic resection procedures were also included in the study. Additionally, patients who had concomitant cryo-ablative procedures for atrial fibrillation and/or closure of the patent foramen oval during the same procedure were included. Exclusion criteria included patients who had additional procedures (other than cryoablation and/or PFO closure) conducted during the mitral valve repair.

### 2.2. Study Type and Data Retrieval

This research is retrospective in nature. Weill Cornell Medicine institutional review board approval number is 1704018121. The data were gathered from the Cardio-Thoracic Information Registry of Weill Cornell Medicine New York Presbyterian Hospital (EPIC, and REDCap for the robotic mitral valve data base).

### 2.3. End Points and Outcomes Definition

In this study, the total operative time was defined as the duration from the initiation of the skin incision to the closure of the incision. This duration specifically excluded the time allocated for anesthesia administration and the initiation and termination of cardiopulmonary bypass (CPB) procedures. Perioperative mortality and morbidity were defined as any operative and early postoperative morbidity and/or mortality occurring within 30 days of the surgery, and all were as defined in the Society of Thoracic Surgeons National Database.

Mitral regurgitation (MR) severity was stratified based on established criteria. Grade +3 MR was categorized as moderately severe, indicating a significant but not yet severe regurgitation, while grade +4 MR was designated as severe, denoting a critical and advanced stage of mitral valve insufficiency.

Our primary outcome is an assessment of the impact of altering the ergonomics of the operating room and the cardiac team on surgeon performance (measured by assessing the operative time). Secondary outcomes include assessment of cardiopulmonary bypass (CPB) and myocardial ischemia times in addition to the rate of conversion to open, i.e., technical proficiency and operative success. Furthermore, we evaluated the effectiveness of robotic cardiac surgery when performed by an experienced surgeon (via assessing the incidence of more than +2 mitral regurgitation at the end of the case and on the predischarge echocardiography in addition to intraoperative blood loss, hospital mortality, new-onset atrial fibrillation, stroke, renal failure, sepsis, the need for ventilator support for more than 24 h, and the need for reoperation due to bleeding, i.e., Patient safety parameters).

### 2.4. Data Statistical Analysis

Continuous variables were presented as median and interquartile range (IQR) and compared using the Mann–Whitney U test, or as mean and standard deviation and compared using the *t* test after testing for normality, while categorical variables were presented as frequency count and percentage and compared across groups using the Chi-square or Fisher’s test, as appropriate.

Learning curve analyses were performed using case sequence numbers, starting with the first robotic case performed in our institute by the index surgeon (S.L.M.). Patients were divided into 2 groups of consecutive patients based on the point of inflection/plateauing to evaluate the influence of growing surgical experience on the total operative time. To clearly identify and depict the relationship between sequence number and continuous responses, we modeled the sequence number and all other variables as additive components using spline smoothing in the semiparametric model [13].

All statistical analyses will be performed using R version 4.2.1 (R Foundation for Statistical Computing) within RStudio. Tableone and ggplot2 R packages were used.

## 3. Results

### 3.1. Patients’ Demographics

Between June 2019 and December 2022, a total of 103 robotic cardiac procedures were performed by a single experienced robotic cardiac surgeon (S.M.K.). The median age of the patients enrolled in the study was 63 years, with 68% being male. The median body mass index (BMI) was 23.87. Table 1 shows the detailed demographics of the studied cohort.

Among the cases studied, 21 patients (20.4%) were identified with preoperative atrial fibrillation (A Fib). Additionally, 9 cases (8.7%) exhibited a history of prior coronary artery disease (CAD), while only one case had a previous myocardial infarction (MI). Eighty-three patients (80%) presented with New York Heart Association (NYHA) class II and III symptoms (57 patients with NYHA class II and 26 patients with NYHA class III, respectively). The median preoperative ejection fraction (EF) was 62 [IQR 55.5–65.0].

### 3.2. Detailed Mitral Pathology

Out of 103 patients, a total of seven cases underwent robotic cardiac procedures for the excision of cardiac tumors, specifically cardiac myxomas. A total of 95 patients (92.2%) exhibited mitral pathology. Notably, all cases presented with either class 3 or class 4 mitral regurgitation (MR) (16.5% vs. 73.8% for class 3 and class 4, respectively). Additionally, two patients demonstrated mitral valve damage resulting from prior endocarditis (Carpentier class 1); one case featured perforation, while the other showed chordae shortening and thickening. Table 1 also showed a detailed description of the mitral pathology.

Furthermore, most cases (90.3%) experienced MR due to degenerative etiology (Carpentier class II). Within this group, 66 cases (64.1%) were attributed to posterior leaflet etiology. 15 cases (14.6%) were diagnosed with bileaflet prolapse (Barlow’s disease), and a smaller subset (8.7%) exhibited anterior leaflet prolapse. The presence of annular dilatation was in 30 cases (29.1%), occurring either in isolation or in conjunction with leaflet pathology (2 cases demonstrated isolated annular dilatation, while in 28 cases, it was associated with other leaflet pathology).

In addition to mitral pathology, various associated cardiac lesions were observed within the studied cohort. Two patients (1.9%) displayed tricuspid regurgitation (TR), another two patients (1.9%) exhibited atrial septal defects (ASD), and three patients (5.8%) presented with patent foramen oval (PFO).

### 3.3. Details of the Used Repair Methods

The predominant repair technique employed in this study was neochordae implantation, which was utilized in 68 patients (66.1%). Within this category, posterior leaflet neochordae account for the majority (58.3%), in contrast to anterior neochordae (7.8%). The second most frequently used repair methods was resection-based techniques (33.9%). Among these, triangular resection was employed in 16.5% of cases, quadrangular resection in 8.7%, and sliding plasty in 8.7% of cases. Alfieri stitches were utilized in only 1.9% of patients. Table 2.

Additionally, nearly all cases underwent annuloplasty, either as a standalone procedure or in combination with other repair methods. The Duran annuloplasty band emerged as the most frequently utilized annuloplasty device (78.6%). Ninety-one percent of patients underwent only one CPB pump run, with only seven patients (6.8%) and two patients (1.9%) undergoing two and three pump runs, respectively. Only 23 cases (22.5%) received an intraoperative blood transfusion, indicating minimal overall blood loss. Table 2.

### 3.4. Assessment of Technical Proficiency

**Figure 1:**
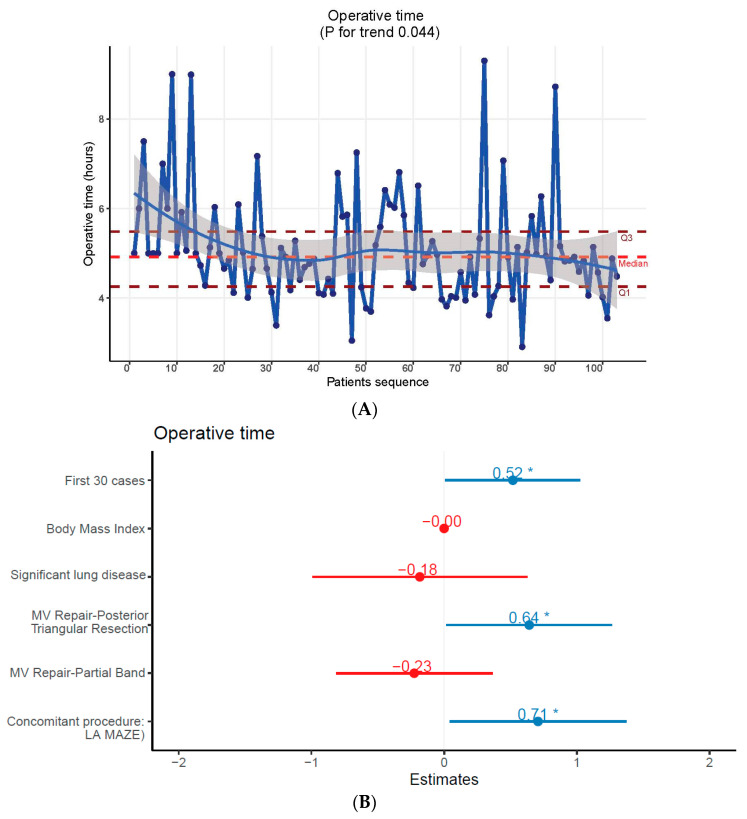
(**A**) operative time trend throughout the study period. (**B**) factors associated with longer operative time on multivariate analysis. Asterix represent the significant *p*-value. Number reflects regression coefficient (Beta). Beta <= 0 was colored in red while beta > 0 was colored in blue.

The operative timeline observed across the study period displayed a distinct inflection point at 30 cases, followed by a plateau. There was a significant reduction from a median of 5.00 h [95% CI: 4.76–6.00] in the initial 30 cases to 4.83 h [4.10–5.27] thereafter, indicating an apparent plateau in the curve (*p* = 0.01). Figure 1A. This trend is suggestive of an adaptation period to the new surgical environment. Subgroup analysis on mitral cases (n = 93) further supported this finding, demonstrating a decrease in ORT from 5.00 h [4.71–5.98] in the first 26 cases to 4.83 h [CI = 4.14–5.30, *p* = *p* = 0.04]. In multivariate analysis, the first 30 cases, resection-based repair techniques, and the inclusion of the LA MAZE procedure as a concomitant surgical intervention emerged as influential factors associated with prolonged ORT. Figure 1B.

**Figure 2:**
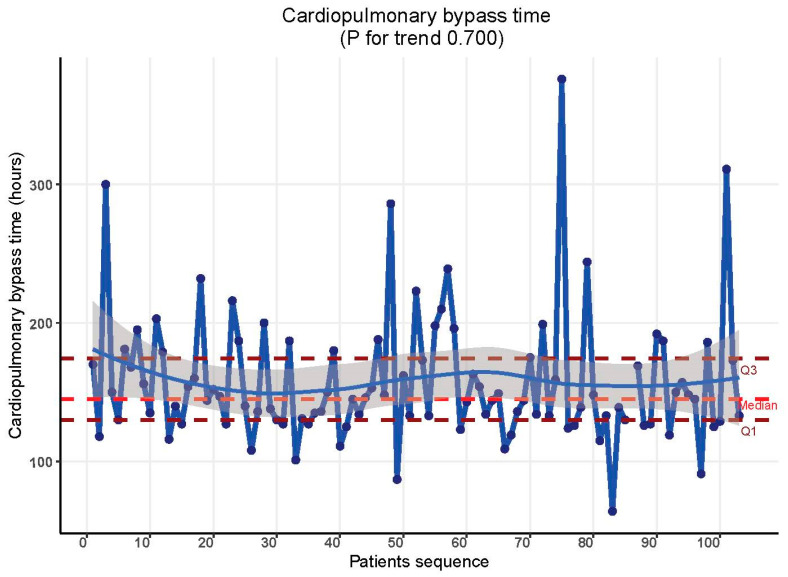
Cardiopulmonary bypass time trend throughout the study period.

**Figure 3:**
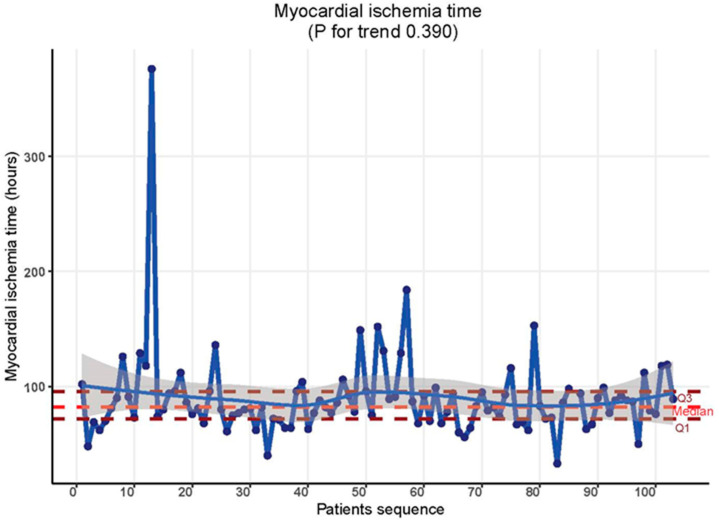
Myocardial ischemia time trend throughout the study period.

In contrast, cardiopulmonary bypass time (CBP) and myocardial ischemia time were not affected by the learning curve phenomenon in this context. Throughout the study timeline, there were no discernible differences in these metrics. CBP time remained consistent at 144.00 min [CI = 128.50, 173.00] in both the initial 30 cases and the subsequent cases (*p* = 0.255). Table 2, Figure 2. Similarly, myocardial ischemia time showed uniformity: 83.00 min [CI = 70.75, 94.25] for the first 30 cases and 81.00 min [CI = 74.25, 96.00] for the subsequent cases (*p* = 0.719). Table 2, Figure 3.

### 3.5. Assessment of Operative Success

Ninety-nine percent of cases (102 patients) were successfully completed as intended, while 1% (1 case) necessitated conversion to an open sternotomy procedure. At the completion of the repair, 95% of cases (97 patients) exhibited either grade +0 or +1 MR, and only 5% of cases (5 patients) had grade +2 MR. Table 2.

### 3.6. Assessment of Patient Safety

Analysis of postoperative complications revealed no significant disparities between the first 30 cases and subsequent cases, indicating that they are not subject to a learning curve phenomenon when surgeons transition between different practice settings. Supplementary Figure S2.

For the overall cohort, 36 cases (35%) experienced an uneventful postoperative course. Eighty-two cases (79.6%) were successfully extubated in the operating room. Only one case (1%) necessitated prolonged ventilatory support exceeding 24 h, while three cases (2.9%) required reintubation during their hospitalization period. The mean duration of the Intensive Care Unit (ICU) stay was 2 days [CI = 2.00, 3.00], with only one case experiencing ICU readmission.

There was no perioperative mortality. Two cases were returned to the operating room due to postoperative bleeding issues. Moreover, only one case required a salvage operation due to valvular dysfunction within the initial 30 days.

In terms of specific complications, 34 cases (33%) developed postoperative A fib, with 25 of them necessitating anticoagulation therapy. Additionally, 13 cases (12.7%) required hospital readmission within 30 days.

During the six-month follow-up period, only two cases developed severe MR, necessitating further intervention.

## 4. Discussion

The healthcare industry operates within a highly intricate ecosystem where expertise and seamless coordination among medical professionals are of utmost importance [14,15]. Within this dynamic environment, a notable phenomenon is the transfer of doctors from one team to another, driven by factors such as professional growth, staffing needs, and advancements in medical technology.

It is well recognized that in medicine, transitions or adaptations can affect health care practitioners’ performance and even patient outcomes. For instance, Scarponi et al. [16] reported a decreased compliance rate when pediatric renal failure patients were transitioned to adult services at the age of 18, and this was notably improved by adopting a model that specifically took into account the additional needs specific to a time of transition. Another study reported that social processes, including alignment professionals working together as cohesive groups, were key factors in establishing well-functioning, successful healthcare endeavors [17]. In Canada, Behruzi et al. reported that lack of interpersonal communication skills among healthcare providers and differences in philosophy and scope of practice affected the quality of patient care [18] and noted that healthcare provider transition is a time associated with risks to that provider, including risks of encountering different goals, changes in work ergonomics, and scopes of practice.

During the transition of healthcare providers, a crucial adjustment period occurs, requiring both the team and the new member (in this situation, the surgeon) to acclimate to each other’s working dynamics [19]. The team must familiarize themselves with the surgeon’s unique approach, preferences, and specialized knowledge. Simultaneously, the transferred surgeon should invest time in understanding the intricacies of the hospital’s protocols, equipment, and procedures. This mutual adaptation process is essential for maintaining the high standards of care and efficiency that patients expect and deserve [20,21].

In this study, our investigation centered around the followings: the impact of altering the ergonomics of the operating room and the cardiac team on surgeon performance, in addition to the evaluation of the effectiveness of robotic cardiac surgery when performed by an experienced surgeon.

Our findings revealed a noteworthy declining trend, followed by a plateau in operative time. After the initial 30 cases, an adaptation period was observed, leading to a plateau in operative time (*p* = 0.01). While this trend was statistically significant, it held limited clinical significance, with operative time reducing marginally from 5.0 [IQR: 4.8–6.0] hours to 4.83 [IQR: 4.1–5.3] hours between the first 30 cases and subsequent cases, respectively. This observation indicates that altering the surgical environment exerts a minimal impact on surgeon performance and operative time, provided both the cardiac team and the surgeon possess adequate experience [22]. Many studies have documented that changing the operating room characteristics might affect overall surgical performance [21,23]. This might also raise the crucial insight that high-volume centers, characterized by standardized protocols, advanced equipment, and consistent operational standards, exhibit striking similarities, shortening the adaptation period. This uniformity in operational procedures across high-volume centers further emphasizes the minimal influence of changes in the surgical environment on surgeon performance when both the team and surgeon have attained the requisite level of proficiency.

Operative success, assessed at the end of MR repair and lack of/minimal conversion to open, along with evaluation of perioperative complications, did not exhibit a learning curve plateau effect. Instead, these outcomes were primarily contingent upon the patient’s performance status and the extensive experience of the surgeon involved.

Several studies have corroborated that the critical decision-making process regarding mitral valve replacement or repair and patients’ outcomes were substantially influenced by the surgeon’s proficiency and their annual caseload [3,4,5,6]. Specifically, it has been established that a reference surgeon should ideally undertake a minimum of 25 index mitral valve procedures within a single calendar year [24]. Moreover, medical centers performing a minimum of 50 index mitral procedures annually are designated as reference centers [24]. This standardized approach has demonstrated a significant impact on the rate of successful repairs and overall perioperative outcomes in national-based (STS-ACS) studies [4,25] and/or institutional-based studies [7,26,27,28].

Our study result reflects the previously mentioned evidence-based facts related to both the surgeon’s and the center’s volume. Notably, we ensured the elimination of bias related to the heterogeneity of surgeons’ experiences and their potential impact on perioperative outcomes. To achieve this, all surgical interventions in our study were exclusively conducted by a single experienced cardiac surgeon, thereby enhancing the reliability and integrity of our study’s results.

Our study possesses inherent limitations, primarily due to its retrospective nature and the utilization of a relatively small sample size within a specific patient group, rendering it susceptible to biases and confounding variables, diminishing its statistical power, and limiting its generalizability to broader populations. However, the study also exhibits a notable strength (the mitigation of bias from heterogeneity in surgeons’ experiences) as all cases were performed by a single experienced surgeon, enhancing the internal validity of the study.

## 5. Conclusions

In this study, total operative time can be expected to decrease after about 30 cases when an experienced robotic surgeon moves between centers. Complications and cross-clamp times are less susceptible to a learning curve phenomenon in such a circumstance, as these depend primarily on the operating surgeon’s level of experience. Patients’ safety was not affected during this transition period. Understanding these dynamics can inform the planning and management of surgical transitions, ensuring optimal patient care and continued improvement in surgical outcomes.

## Figures and Tables

**Table 1:** Criteria of included patients.

	**Overall**	**After**	**First 30 Cases**	***p***
N	103	73	30	
Age (years (median [IQR])	63.00 [56.50, 69.00]	62.00 [53.00, 68.00]	65.50 [58.25, 69.00]	0.235
Gender (Males %)	70 (68.0)	52 (71.2)	18 (60.0)	0.38
Diagnosis				
MR (%)	93 (90.3)	67 (91.8)	26 (86.7)	0.667
TR (%)	2 (1.9)	0 (0.0)	2 (6.7)	0.149
Cardiac Tumor (Atrial myxoma (%))	7 (6.8)	4 (5.5)	3 (10.0)	0.691
ASD (%)	2 (1.9)	1 (1.4)	1 (3.3)	1
Afib (%)	9 (8.7)	5 (6.8)	4 (13.3)	0.5
Heart Failure (%)	1 (1.0)	0 (0.0)	1 (3.3)	0.644
PFO (%)	3 (2.9)	0 (0.0)	3 (10.0)	0.036
Other (%)	6 (5.8)	3 (4.1)	3 (10.0)	0.486
Body Mass Index (median [IQR])	23.87 [22.02, 26.49]	24.34 [22.07, 26.18]	23.77 [21.94, 26.83]	0.925
Prior HTN (%)	47 (45.6)	32 (43.8)	15 (50.0)	0.724
Prior DM (%)	3 (2.9)	3 (4.1)	0 (0.0)	0.63
Prior HLD (%)	41 (39.8)	26 (35.6)	15 (50.0)	0.257
Prior CAD (%)	9 (8.7)	7 (9.6)	2 (6.7)	0.926
Prior Peripheral artery disease (%)	1 (1.0)	1 (1.4)	0 (0.0)	1
Prior Atrial Fibrillation (%)	21 (20.4)	12 (16.4)	9 (30.0)	0.199
Prior Atrial Flutter (%)	3 (2.9)	1 (1.4)	2 (6.7)	0.419
Prior MI (%)	1 (1.0)	1 (1.4)	0 (0.0)	1
Prior Significant lung disease (%)	9 (8.7)	6 (8.2)	3 (10.0)	1
Prior Liver disease (%)	1 (1.0)	1 (1.4)	0 (0.0)	1
Prior CVA (%)	3 (2.9)	2 (2.7)	1 (3.3)	1
Prior Smoking (%)	27 (26.2)	19 (26.0)	8 (26.7)	1
NYHA class (%)				
1	20 (19.4)	16 (21.9)	4 (13.3)	0.507
2	57 (55.3)	38 (52.1)	19 (63.3)	
3	26 (25.2)	19 (26.0)	7 (23.3)	
Ejection fraction, ___% (median [IQR])	62.00 [55.50, 65.00]	61.00 [58.00, 65.00]	65.00 [54.00, 67.00]	0.881
AI (%)				
0	72 (69.9)	49 (67.1)	23 (76.7)	0.428
1	29 (28.2)	23 (31.5)	6 (20.0)	
2	2 (1.9)	1 (1.4)	1 (3.3)	
MR (%)				
0	8 (7.8)	5 (6.8)	3 (10.0)	0.294
1	1 (1.0)	0 (0.0)	1 (3.3)	
2	1 (1.0)	1 (1.4)	0 (0.0)	
3	17 (16.5)	10 (13.7)	7 (23.3)	
4	76 (73.8)	57 (78.1)	19 (63.3)	
TR (%)				
0	50 (48.5)	34 (46.6)	16 (53.3)	0.224
1	48 (46.6)	35 (47.9)	13 (43.3)	
2	4 (3.9)	4 (5.5)	0 (0.0)	
3	1 (1.0)	0 (0.0)	1 (3.3)	
Etiology of MV disease				
unknown (%)	1 (1.0)	1 (1.4)	0 (0.0)	1
degenerative (%)	91 (88.3)	65 (89.0)	26 (86.7)	0.997
Other (%)	2 (1.9)	2 (2.7)	0 (0.0)	0.897
Barlows Disease (%)	15 (14.6)	12 (16.4)	3 (10.0)	0.593
Prior endocarditis with damage to MV (%)	2 (1.9)	2 (2.7)	0 (0.0)	0.897
MV lesions				
posterior leaflet prolapse (%)	66 (64.1)	48 (65.8)	18 (60.0)	0.744
anterior leaflet prolapse (%)	9 (8.7)	4 (5.5)	5 (16.7)	0.149
bi-leaflet prolapse (%)	15 (14.6)	11 (15.1)	4 (13.3)	1
elongated/ruptured chords (%)	72 (69.9)	50 (68.5)	22 (73.3)	0.802
annular dilation (%)	30 (29.1)	27 (37.0)	3 (10.0)	0.012
chordal thickening and shortening (%)	1 (1.0)	1 (1.4)	0 (0.0)	1
calcified leaflets (%)	1 (1.0)	0 (0.0)	1 (3.3)	0.644
leaflet perforation (%)	1 (1.0)	1 (1.4)	0 (0.0)	1
None (%)	5 (4.9)	4 (5.5)	1 (3.3)	1
Other (%)	13 (12.6)	11 (15.1)	2 (6.7)	0.401
Carpentier MR Classification (%)				
Type I	2 (1.9)	1 (1.4)	1 (3.3)	1
Type II	93 (90.3)	67 (91.8)	26 (86.7)	0.667
Type IIIa	0 (0.0)	0 (0.0)	0 (0.0)	NA
Type IIIb	0 (0.0)	0 (0.0)	0 (0.0)	NA
N/A	6 (5.8)	3 (4.1)	3 (10.0)	0.486

No cases with MS, CRI, hemodialysis, carotid stenosis, TIA, prior cardiac surgery, Rheumatic heart disease. Regarding etiology, there were no cases with rheumatic, acute/chronic ischemic MR, acute endocarditis, rheumatic, ischemic, post-infarction, ischemic chronic endocarditis, HOCM, trauma, congenital, prior MV intervention, or SAM. No cases with pap muscle rupture, commissural fusion, Restrictive anterior/posterior leaflet.

**Table 2:** Operative details and outcomes.

	**Overall**	**After**	**First 30 Cases**	***p***
Operative details and outcomes	103	73	30	
Operative time (mean (SD))	5.10 (1.21)	4.93 (1.15)	5.51 (1.28)	0.026
Operative time (median [IQR])	4.92 [4.26, 5.48]	4.83 [4.10, 5.27]	5.00 [4.76, 6.00]	0.01
No. of pump runs (%)				
1	94 (91.3)	66 (90.4)	28 (93.3)	0.656
2	7 (6.8)	5 (6.8)	2 (6.7)	
3	2 (1.9)	2 (2.7)	0 (0.0)	
Operations performed:				
MV repair (%)	93 (90.3)	67 (91.8)	26 (86.7)	0.667
TV repair (%)	4 (3.9)	2 (2.7)	2 (6.7)	0.707
ASD closure (%)	3 (2.9)	2 (2.7)	1 (3.3)	1
Pulmonary vein isolation (%)	1 (1.0)	1 (1.4)	0 (0.0)	1
LA MAZE (%)	15 (14.6)	9 (12.3)	6 (20.0)	0.487
LA appendage closure (%)	6 (5.8)	3 (4.1)	3 (10.0)	0.486
PFO closure (%)	7 (6.8)	4 (5.5)	3 (10.0)	0.691
Cardiac tumor removal (%)	7 (6.8)	4 (5.5)	3 (10.0)	0.691
Other (%)	6 (5.8)	3 (4.1)	3 (10.0)	0.486
If MV Repair:				
Posterior Triangular Resection (%)	17 (16.5)	12 (16.4)	5 (16.7)	1
Posterior Quadrangular Resection (%)	9 (8.7)	8 (11.0)	1 (3.3)	0.389
Sliding Plasty (%)	9 (8.7)	9 (12.3)	0 (0.0)	0.103
Neochords to Posterior Leaflet (%)	60 (58.3)	43 (58.9)	17 (56.7)	1
Anterior Leaflet Neochords (%)	8 (7.8)	5 (6.8)	3 (10.0)	0.891
Partial Band (%)	81 (78.6)	58 (79.5)	23 (76.7)	0.961
Complete Flexible Ring (%)	2 (1.9)	1 (1.4)	1 (3.3)	1
Complete Rigid/Semi-Rigid Ring (%)	1 (1.0)	1 (1.4)	0 (0.0)	1
Partial Semi-Rigid Band (%)	8 (7.8)	7 (9.6)	1 (3.3)	0.501
Annular Reconstruction (%)	1 (1.0)	0 (0.0)	1 (3.3)	0.644
Patch closure of perforation (%)	1 (1.0)	1 (1.4)	0 (0.0)	1
Alfieri Stitch (%)	2 (1.9)	1 (1.4)	1 (3.3)	1
Commisurroplasty (Magic Stitch) (%)	10 (9.7)	7 (9.6)	3 (10.0)	1
MV cleft closure (%)	33 (32.0)	23 (31.5)	10 (33.3)	1
Other (%)	18 (17.5)	8 (11.0)	10 (33.3)	0.015
Cardiopulmonary Bypass Time (median [IQR])	145.00 [130.00, 174.50]	144.00 [128.50, 173.00]	144.00 [128.50, 173.00]	0.255
Aortic Cross-clamp time (median [IQR])	82.00 [72.00, 95.75]	83.00 [70.75, 94.25]	81.00 [74.25, 96.00]	0.719
Did the patient receive blood products in the OR? (%)	23 (22.5)	17 (23.6)	6 (20.0)	0.891
MR grade at the end of the case (%)				
0	65 (63.7)	51 (69.9)	14 (48.3)	0.123
1	32 (31.4)	19 (26.0)	13 (44.8)	
2	5 (4.9)	3 (4.1)	2 (6.9)	
Extubation in in the OR (%)	82 (79.6)	56 (76.7)	26 (86.7)	0.384
Conversion to open procedure (%)	1 (1.0)	1 (1.4)	0 (0.0)	1
Postoperative blood product (%)	16 (15.5)	12 (16.4)	4 (13.3)	0.924
Postoperative complications				
Return to OR for bleeding (%)	2 (1.9)	2 (2.7)	0 (0.0)	0.897
Prolonged Ventilation >24 h (%)	1 (1.0)	1 (1.4)	0 (0.0)	1
Reintubated during hospitalization (%)	3 (2.9)	1 (1.4)	2 (6.7)	0.419
New/Acute Renal Failure (%)	2 (1.9)	1 (1.4)	1 (3.3)	1
Groin Infection (%)	1 (1.0)	0 (0.0)	1 (3.3)	0.644
Groin Lymphocele (%)	4 (3.9)	2 (2.7)	2 (6.7)	0.707
Need for IABP (%)	1 (1.0)	0 (0.0)	1 (3.3)	0.644
Readmit to ICU (%)	1 (1.0)	0 (0.0)	1 (3.3)	0.644
Reoperation for valvular dysfunction within 30 days (%)	1 (1.0)	0 (0.0)	1 (3.3)	0.644
Pneumonia (%)	2 (1.9)	1 (1.4)	1 (3.3)	1
Pleural Effusion requiring drainage (%)	7 (6.8)	6 (8.2)	1 (3.3)	0.642
DVT (%)	1 (1.0)	0 (0.0)	1 (3.3)	0.644
Pneumothorax requiring intervention (%)	2 (1.9)	1 (1.4)	1 (3.3)	1
Tamponade, surgical intervention (%)	1 (1.0)	1 (1.4)	0 (0.0)	1
Aortic Dissection (%)	1 (1.0)	1 (1.4)	0 (0.0)	1
Atrial Fibrillation (%)	34 (33.0)	21 (28.8)	13 (43.3)	0.231
Uneventful post operative course (%)	36 (35.0)	25 (34.2)	11 (36.7)	0.995
If re-exploration for bleeding:				
VATs or Robotic (%)	1 (1.0)	1 (1.4)	0 (0.0)	1
Mini-Thoracotomy (%)	1 (1.0)	1 (1.4)	0 (0.0)	1
Anti-coagulation required in AF (%)	25 (24.3)	15 (20.5)	10 (33.3)	0.262
Did the patient survive 30 day or discharge whichever is longer? (%)	101 (99.0)	72 (100.0)	29 (96.7)	0.65
Readmitted within 30 days? (%)	13 (12.7)	9 (12.5)	4 (13.3)	1
ICU stay (days) (median [IQR])	2.00 [2.00, 3.00]	2.00 [2.00, 3.00]	2.00 [2.00, 4.00]	0.293
Last follow-up status (Alive (%))	103 (100.0)	73 (100.0)	30 (100.0)	NA
MR degree at last follow up (%)				
no	66 (64.1)	45 (61.6)	21 (70.0)	0.082
mild +1	12 (11.7)	7 (9.6)	5 (16.7)	
moderate	1 (1.0)	0 (0.0)	1 (3.3)	
severe	2 (1.9)	1 (1.4)	1 (3.3)	
trace	22 (21.4)	20 (27.4)	2 (6.7)	

No cases underwent Anterior Leaflet Resection or Commissurotomy, Leaflet plication, Anterior or posterior Leaflet Augmentation, Folding Plasty, or Posterior Annular Decalcification. No cases had TV replacement, Bi Atrial MAZE, Stem Cell implant, VSD Repair, Septal Myectomy for HOCM, Robotic MIDCAB, TECAB, Reconstruction of Damaged Leaflet, and Complete Reconstruction of Valve with Tissue. No cases with CVA, CVA with neuro deficit >7 days), Trach during hospitalization, Renal Failure requiring New Dialysis, Chest Wall Incision Infection, Chest Wall Incision Infection, sepsis, Limb loss or Limb ischemic complications, PE, Positive blood cultures, Phrenic nerve injury, Cardiac Arrest, VA ECMO, VV ECMO, Multi-system organ failure, Hemoperitoneum, Liver injury, Diaphragm injury. No cases with re-exploration for bleeding through Sternotomy, Laparotomy, or Laparoscopy.

## Data Availability

The data are not publicly available due to commercial confidentiality, as they contain information that could compromise the privacy of research participants.

## Supplementary Materials

The following supporting information can be downloaded at: https://www.mdpi.com/article/10.3390/jcdd11030081/s1, Figure S1: The robotic team ergonomics at Weill Cornell Medicine; Figure S2: Postoperative outcomes.
