# Supplementary material for: Transferring Surgical Expertise: Analyzing the Learning Curve of Robotic Cardiac Surgery Operative Time Reduction When Surgeon Moves from One Experienced Center to Another
Source: J Cardiovasc Dev Dis. 2024 Feb 29;11(3):81. doi: 10.3390/jcdd11030081 (PMC10971129; doi:10.3390/jcdd11030081)

Supplementary Figure S1: the robotic team ergonomics at Weill Cornell Medicine.

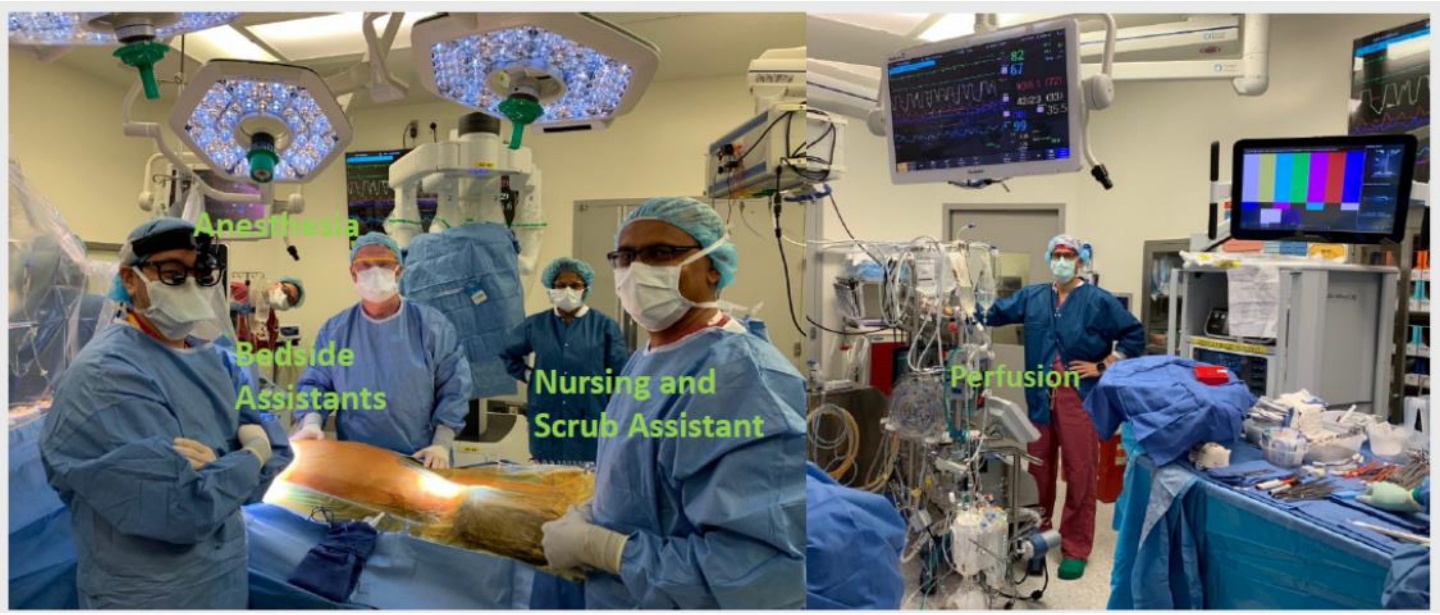

Supplementary Figure S2: Postoperative outcomes

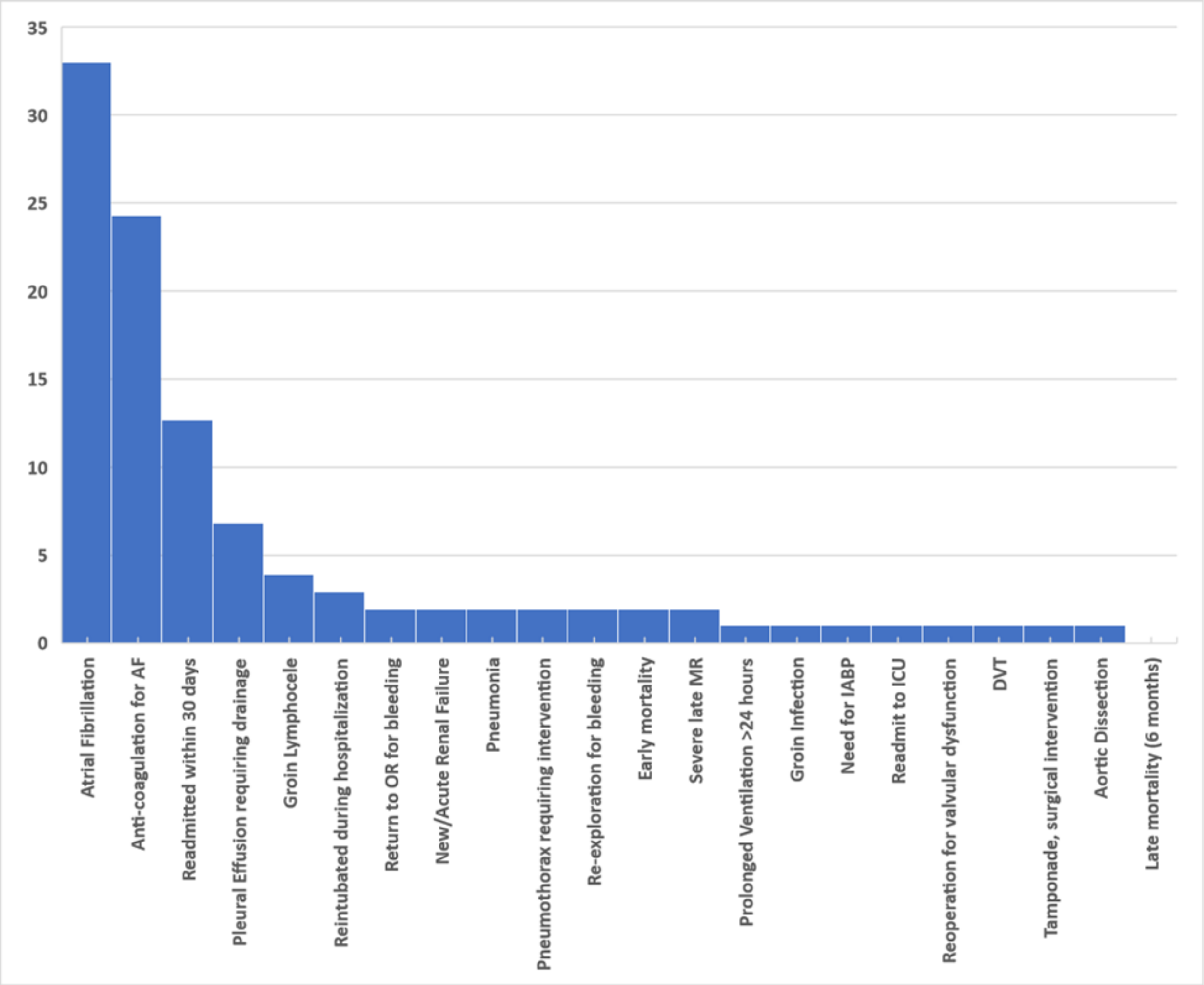

Supplement: Supplementary file 1 [file jcdd-11-00081-s001.zip › jcdd-2863102-supplementary.pdf]
